# Increased number of negative lymph nodes is associated with improved survival outcome in node positive gastric cancer following radical gastrectomy

**DOI:** 10.18632/oncotarget.9041

**Published:** 2016-04-27

**Authors:** Rong-liang Shi, Qian Chen, Jun Bing Ding, Zhen Yang, Gaofeng Pan, Daowen Jiang, Weiyan Liu

**Affiliations:** ^1^ Department of General Surgery, Minhang Hospital, Fudan University, Shanghai, People's Republic of China; ^2^ Department of Head and Neck Surgery, Fudan University Shanghai Cancer Center, Shanghai, People's Republic of China; ^3^ Department of Oncology, Shanghai Medical College, Fudan University, Shanghai, People's Republic of China; ^4^ Department of Thoracic Surgery, Minhang Hospital, Fudan University, Shanghai, People's Republic of China

**Keywords:** gastric cancer, negative lymph node, SEER

## Abstract

The concept of negative lymph node (NLN) counts has recently attracted attention as a prognostic indicator in various cancer. However, the correlation between NLN counts and patient prognosis in the setting of gastric cancer is not fully studied. Surveillance, Epidemiology, and End Results Program (SEER)-registered gastric cancer patients were used for analysis in this study. Clinicopathological characteristics, including race, age, gender, and tumor stage, grade, and cause specific survival were collected. Univariate and multivariate Cox proportional hazards model were used to assess the risk factors for survival. As results, X-tile plots identified 3 and 9 as the optimal cutoff value to divide the patients into high, middle and low risk subsets in terms of cause specific survival, and NLN was validated as independently prognostic factor in mulivariate Cox analysis (*P* < 0.001). Further analysis showed that NLN was a prognosis factor in each N stage. Collectively, our study results firmly demonstrated that the number of NLNs was an independent prognostic factor for gastric cancer patients, and together with the N stage, it could provide more accurate prognostic information than the N stage alone.

## INTRODUCTION

Gastric cancer (GC) is one of major cause of cancer related death worldwide because of its poor prognosis [1]. Surgical resection is the primary therapy option that offers the only curative chance for GC and lymph node status was a strong predictor for patients with GC after surgical resection. Although it has been shown that a 15-node minimum has been endorsed as a consensus standard for proper tumor stage and associated with a good survival outcome in patients treated with gastrectomy for GC [2]; However, a subgroup of them die of recurrence and metastases. In fact, the node-positive patients with GC are heterogeneous and the prognosis of these patients cannot be stratified by the node-stage only [3-5]. In view of these, pinpoint of prognostic factors, especially a common clinicopathological factor, for recurrence and metastasis would allow to find patients at high risk that may benefit from adjuvant chemotherapy or intensive care, usually proposed to node-positive GC patient only [6]. The concept of negative lymph node (NLN) counts has recently attracted attention as a prognostic indicator in colon [7, 8], gastric [9], esophageal [10], and cervical [11]. However, the correlation between NLN counts and patient prognosis in the setting of GC is not fully studied. Therefore, the aims of this retrospective study is to explore the effect of NLN counts on the long-term survival outcome in patients with GC after surgical resection. GC.

In the present article, the Surveillance, Epidemiology, and End Results (SEER)-registered database for GC is analyzed, aimed to investigate role of NLN count on node-positive GC patients' survival outcomes after surgical treatment.

## RESULTS

### SEER database patient characteristics

In our 9-year study period, a total of 6,177 eligible GC patients were enrolled in the current study. Figure S1 depicts the flow chart of the study. The median follow up time was 27 months (0-127 months). The median age at diagnosis was 67 years (range, 21–99 years). Median survival time was 27 months with the 5-year GC-cause specific survival rate (GCSS) was 36.0%. Data from 3,124 (50.6%) patients were death. The mean number of LNs examined was 17.72 (range, 1–89), the median positive LN (PLN) count was 5.72 (range, 1–68), and the median NLN was 11.99(0-86). Patient demographics and baseline are listed in Table 1. In this setting, N stage was correlated with the number of dissected LN count, but had a significant reversely impact on NLNs (*P* < 0.001). A significant relationship was also found between NLNs proportion and retrieved nodes (*P* < 0.001). Meanwhile, Correlations between the number of NLNs and positive lymph node were weak or negligible (*r* = −0.108) [12].

**Table 1:** Demographic and tumor characteristics of patients with node positive gastric cancer

	Subgroup						χ^2^ Value	*P* Value
	N1		N2		N3			
	n=2221		n=2059		n=1897			
Characteristic	No.	%	No.	%	No.	%		
Sex							2.308	0.315
male	1408	63.4%	1346	65.4%	1238	65.3%		
female	813	36.6%	713	34.6%	659	34.7%		
Age							17.244	<0.001
≤60	662	29.8%	718	34.9%	666	35.1%		
>60	1559	70.2%	1341	65.1%	1231	64.9%		
Race							35.363	<0.001
Caucasian	1491	67.1%	1377	66.9%	1194	62.9%		
Black	297	13.4%	305	14.8%	243	12.8%		
Other*	433	19.5%	377	18.3%	460	24.3%		
Pathological grading							98.763	<0.001
High/Moderate	740	33.3%	528	25.6%	380	20.0%		
Poor/Anaplastic	1412	63.6%	1483	72.0%	1462	77.1%		
Unknown	69	3.1%	48	2.3%	55	2.9%		
Histotype							16.938	<0.001
Adenocarcinoma	1720	77.4%	1505	73.1%	1483	78.2%		
Mucinous/Signet ring cell	501	22.6%	554	26.9%	414	21.8%		
T stage							434.932	<0.001
T1	338	15.2%	152	7.4%	44	2.3%		
T2	372	16.7%	212	10.3%	118	6.2%		
T3	961	43.3%	1002	48.7%	883	46.5%		
T4	550	24.8%	693	33.7%	852	44.9%		
No. of LNs dissected	14.72(1-87)		16.57(3-89)		22.47(7-88)			<0.001**
No. of positive LNs	1.43(1-2)		4.23(3-6)		12.35(7-68)			<0.001**
No. of negative LNs	13.29(0-86)		12.34(0-84)		10.09(0-76)			<0.001**

* Other includes American Indian/Alaska native, Asian/Pacific Islander, and unknown.

** One-way ANOVA analysis

### Identification of cutoff points for the NLN retrieved in lymph node positive patients in the SEER database

**Figure 1:**
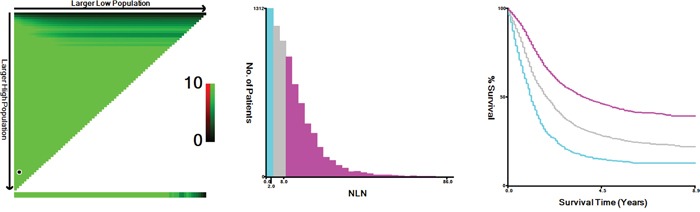
X-tile analysis of survival data from the SEER registry X-tile analysis was performed using data from SEER database, which were equally divided into training and validation sets. X-tile plots of the training sets are shown in the *left panels*, with plots of matched validation sets shown in the *smaller inset*. The optimal cut-point highlighted by the *black circle* in the *left panels* is shown on a histogram of the entire cohort (*middle panels*), and a Kaplan-Meier plot (*right panels*). *P* values were determined using the cutoff point defined in the training set and applying it to the validation set. *Figure 1* shows the optimal cutoff point for the lymph node positive patients (number 3 and 9, χ2=490.428, *P* < 0.001).

We first treated NLN count as a continuous variable, and it was validated as a significant prognostic factor by univariate Cox analysis (*χ* ^2^ = 363.387, *P* < 0.001). Next, X-tile plots were constructed and the maximum χ2 log-rank value of 490.428 (*P* < 0.001) was produced, applying 3 and 9 as the optimal cutoff value to divide the cohort into high, middle and low risk subsets in terms of GCSS. (Figure 1). There was an absolute 29.7% improvement in 5-year GCSS if ≥9 NLNs were analyzed compared with those who had <3.

### Impact of the number of NLNs on GCSS in the SEER database

The number of NLNs and other clinicopathological factors, including age (*P* < 0.001), race (*P* < 0.001), poor and undifferentiated tumor grade (*P* < 0.001), advanced T stage (*P* < 0.001), and higher N stage (*P* < 0.001) were significant correlated with poor survival outcome on univariate analysis (Table 2). A reduced model was used in the multivariate Cox analysis as previous described [13]. Multivariate Cox regression analysis demonstrated that age, race, grade, T stage, N stage, and NLN group were independent predictors of GCSS and a higher number of NLNs were found to have a reduced risk of death on survival (NLN number 3-8, hazard ratio [HR] 0.680; 95% confidence interval [CI] 0.617-0.750; ≥9, HR 0.452; 95% CI 0.411-0.496. Table 2).

**Table 2:** Univariate and multivariate survival analyses for evaluating the influence of the number of NLNs retrieved on GCSS in node positive gastric cancer

Variable	5-year RCCS	Univariate analysis		Multivariate analysis	
		Log rank χ^2^ test	P	HR(95%CI)	P
Sex		0.372	0.542		NI
Male	36.2%				
Female	36.2%				
Age		69.746	<0.001		<0.001
≤60	41.7%			Reference	
>60	33.4%			1.486(1.375-1.605)	
Race		44.286	<0.001		<0.001
Caucasian	34.2%			Reference	
Black	33.2%			1.035(0.934-1.147)	0.508
Others	44.7%			0.712(0.648-0.783)	<0.001
Grade		39.699	<0.001		0.002
High/Moderate	42.7%			Reference	
Poor/Anaplastic	33.9%			1.165(1.071-1.268)	<0.001
Unknown	33.3%			1.127(0.908-1.398)	0.278
Histotype		0.032	0.859		NI
Adenocarcinoma	36.4%				
Mucinous/signet ring cell	35.5%				
T Stage		64.721	<0.001		<0.001
T1	64.2%			Reference	
T2	55.7%			1.083(0.882-1.331)	0.448
T3	36.4%			1.805(1.523-2.139)	<0.001
T4	22.6%			2.425(2.042-2.881)	<0.001
No. of NLNs		490.428	<0.001		<0.001
0-2	16.4%			Reference	
3-8	29.0%			0.680(0.617-0.750)	<0.001
≥9	46.1%			0.452(0.411-0.496)	<0.001
N stage		315.020	<0.001		<0.001
N1	48.8%			Reference	
N2	35.9%			1.282(1.171-1.403)	<0.001
N3	21.6%			1.745(1.593-1.912)	<0.001

NI: not included in the multivariate survival analysis.

### Subgroup analysis for evaluating the effect of NLN counts according to N stage in the SEER database

**Figure 2:**
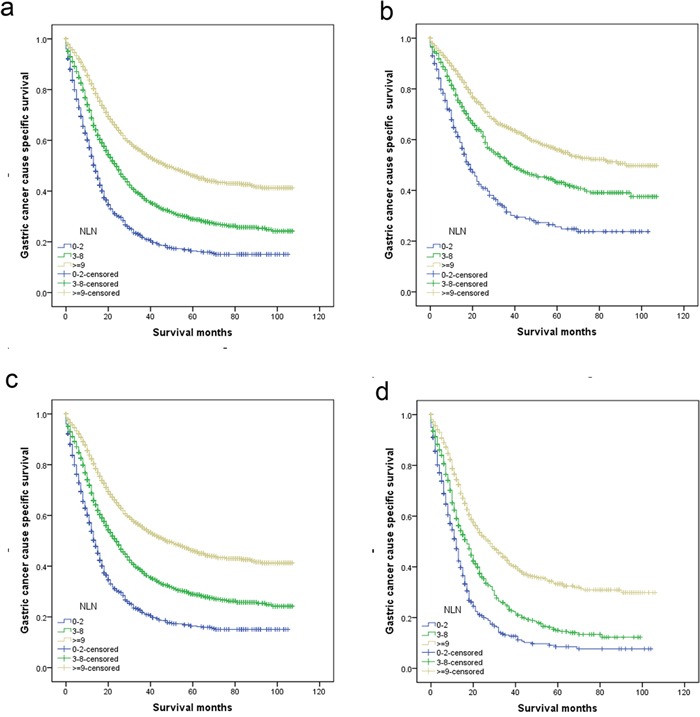
Log-rank tests of cause specific comparing those who had ≥9, 3-8, and <3 negative lymph nodes for **a.** all stage: χ^2^ = 490.428, *P* < 0.001; **b.** N1 stage: χ^2^ = 121.351, *P* < 0.001; **c.** N2 stage: χ^2^ = 118.978, *P* < 0.001; **d.** N3 stage: χ^2^ = 163.051, *P* < 0.001.

We then made further analysis of the effects of NLN on survival in each N stage. After stratifying patients by N stage, NLN counts were validated as independently prognostic factor in each N stage on both univariate and multivariate analysis (*P* < 0.001); For GC patients with N1 stage, there was an absolute 30.2% improvement in 5-year GCSS if ≥9 NLNs were analyzed compared with to those who had <3 (*P* < 0.001). Similarly, in patients with N2 and N3 stage cancer, there were absolute 24.8% improvement in 5-year GCSS if ≥9 NLN were analyzed compared with to those who had <3 (*P* < 0.001). (Table 3, Figure 2). Of particular importance, the 5-year GCSS for N3 patients with ≥9 NLN was better than N1 and N2 patients with 0-2 NLN counts (Table 3).

**Table 3:** Univariate and multivariate analyses of NLN count on GCSS based on different cancer stage

Variable	5-year RCCS	Univariate analysis		Multivariate analysis	
		Log rank χ^2^ test	P	HR(95%CI)	P
**N Stage**					
**N1 stage**					
No. of NLNs		121.351	<0.001		<0.001
0-2	25.7%			Reference	
3-8	43.1%			0.640(0.526-0.779)	<0.001
≥9	55.9%			0.440(0.367-0.528)	<0.001
**N2 stage**					
No. of NLNs		118.978	<0.001		<0.001
0-2	19.1%			Reference	
3-8	29.2%			0.697(0.584-0.831)	<0.001
≥9	43.9%			0.471(0.397-0.558)	<0.001
**N3 stage**					
No. of NLNs		163.051	<0.001		<0.001
0-2	8.5%			Reference	
3-8	15.0%			0.684(0.591-0.791)	<0.001
≥9	33.3%			0.436(0.376-0.505)	<0.001

*P*-values refer to comparisons between two groups and were adjusted for age, race, pathological grading, and tumor stage as covariates.

## DISCUSSION

Despite advances in early diagnosis, operative technique, and adjuvant therapies, radical gastrectomy remains the only potentially curative treatment for GC. However, even after surgical management, the 5-year overall survival rate is only about 20% in series from the United States [14]. It is generally accepted that gastrectomy with dissection of the regional lymph nodes would improve the 5-year survival of patients with GC cancer [15, 16]. At least of 15 lymph nodes should be retrieval from surgical specimens for accurate postoperative stage according to the 7^th^ edition tumor-node-metastasis (TNM) staging system for GC [5, 17, 18]. The N category is defined by the number of positive nodes is one of the most important predictors of survival outcomes in patients with GC and has been included as a staging parameter in the 7^th^ edition UICC/AJCC TNM staging system for GC [5, 18]. However, the node-positive patients GC are heterogeneous and the prognosis of these patients cannot be accurately stratified by the node-stage system alone [5]. The number of NLNs has been confirmed as an independent prognosis factor in colon [7, 8], gastric [9], esophageal [10], et al. Taking account of these considerations, the aim of present study was to investigate the prognostic value of NLN count in the patients with node-positive GC. In this large population based study, our results demonstrated that the NLNs count was an independent prognosis factor for GC, and even in subgroup analysis of N1, N2, and N3 stage patients, increased numbers of NLNs were associated with improved GCSS. The number of NLNs had a weak or negligible correlation with PLN counts, which means that it was a predictor independent of current metastatic LNs count-based staging.

Several hypotheses have been proposed to explain the underlying mechanism for the relationship between the number of NLNs and survival. The first hypothesis involves stage-migration. The extent of lymphadenectomy for curative GC gastrectomy has been debated for several decades; however it is generally accepted that more radical surgery is associated with better lymph node examination [9, 19]. Our study indicated that there was a significant relationship between the number of total LN and NLN counts; the more LN examined, the more likely that it reflects the true stage, and lower nodal counts may increase the risk of under-staging, and a higher number of LNs examined was linked with more accurate N stage classification. Second, the surgeon is a technician. Theoretically, a great number of retrieved NLNs indicate an authoritative surgical curability and quality of surgical care or pathology. The isolated tumor cellsin NLN are though as the main source that could lead to poor survival outcomes of GC patients [20-22]. Third, NLN may simply reflect a host lymphocytic reaction to the tumor [23], and lymphocytic reaction to tumor cells has been associated with longer survival in cancer patients [24, 25].

The results of this study must be interpreted in the light of certain limitations. First, the quality of surgical care or pathological technique may affect LN harvest, we cannot adjust these in our analysis. Second, distant lymph node metastases always classified as distant metastases and therefore the surgery were regarded as palliative resection, and should be excluded from this study. But for SEER data lacks such information, we cannot adjust for this. Third. SEER database lacks information of therapy options (radical resection or palliative therapy), subsequent therapy, co-morbidities and recurrence, which may also impact patients' survival outcome.

Despite these potential limitations, our study results firmly demonstrated that NLNs counts was an independent prognostic predictor for patients with GC after gastrectomy, and together with the N stage, it provides more accurate prognostic information than the N stage alone.

## MATERIALS AND METHODS

### Patient selection in the SEER database

Data were obtained from the publicly available version of the SEER database, which consisted of 18 population-based cancer registries covering approximately 28% of the population of the United States. The National Cancer Institute's SEER*Stat software (Surveillance Research Program, National Cancer Institute SEER*Stat software, www.seer.cancer.gov/seerstat; Version 8.1.5) was used to access the database.

We searched for patients diagnosed between 2004 and 2012 with single primary GC. Histological types were limited to adenocarcinoma, mucinous adenocarcinoma, and signet ring cell carcinoma. Patients were excluded if age at diagnosis was less than 18 years, had more than one primary cancer but the gastric wasn't the first one, had no surgical resection or palliative resection (patients with distant metastasis), had undefined TNM stage, had unknown cause of death or unknown survival months.

Patients' demographic and clinicopathological variables, including age, sex, race, tumor grade, histologic type, T or N stage, primary site, tumor metastatic status, treatment type, reginal lymph node retrieval, reginal lymph node metastasis, et al, were retrieved from the SEER database. The primary endpoint in this study was GC cause specific survival (GCSS), defined as the period from diagnosis to death due to GC. Data of patients who died from other causes or who were alive on the date of their last follow-up were censored.

### Statistical analyses

The LNs cutoff points were analyzed using the X-tile program (http://www.tissuearray.org/rimmlab/), which identified the cutoff with the minimum P values from log-rank χ^2^ statistics for the categorical LNs in terms of survival [26, 27]. A comparison of the categorical variables between N stage subgroups was conducted using Pearson's χ2 test. Continuous variables were compared using the Mann-Whitney *U* test. The Kaplan-Meier method was used to calculate the actual survival rate and to plot survival curves, followed by the log-rank test for clinical and histological variables. The Cox proportional hazard regression model was used to identify the variables that could independently influence survival in GC. Hazard ratios (HRs) and 95% confidence intervals were calculated, with an HR of <1.0 indicating survival benefit. All statistical analyses were performed using SPSS ver.19.0 (SPSS Inc., Chicago, IL), and a value of *P* < 0.05 indicated statistical significance. All tests were 2 sided with *p* < 0.05 defined as statistically significant.

## SUPPLEMENTARY FIGURE


