# Supplementary figures and images for: Increased number of negative lymph nodes is associated with improved survival outcome in node positive gastric cancer following radical gastrectomy
Source: Oncotarget. 2016 Apr 27;7(23):35084–91. doi: 10.18632/oncotarget.9041 (PMC5085211; doi:10.18632/oncotarget.9041)

## SUPPLEMENTARY FIGURE

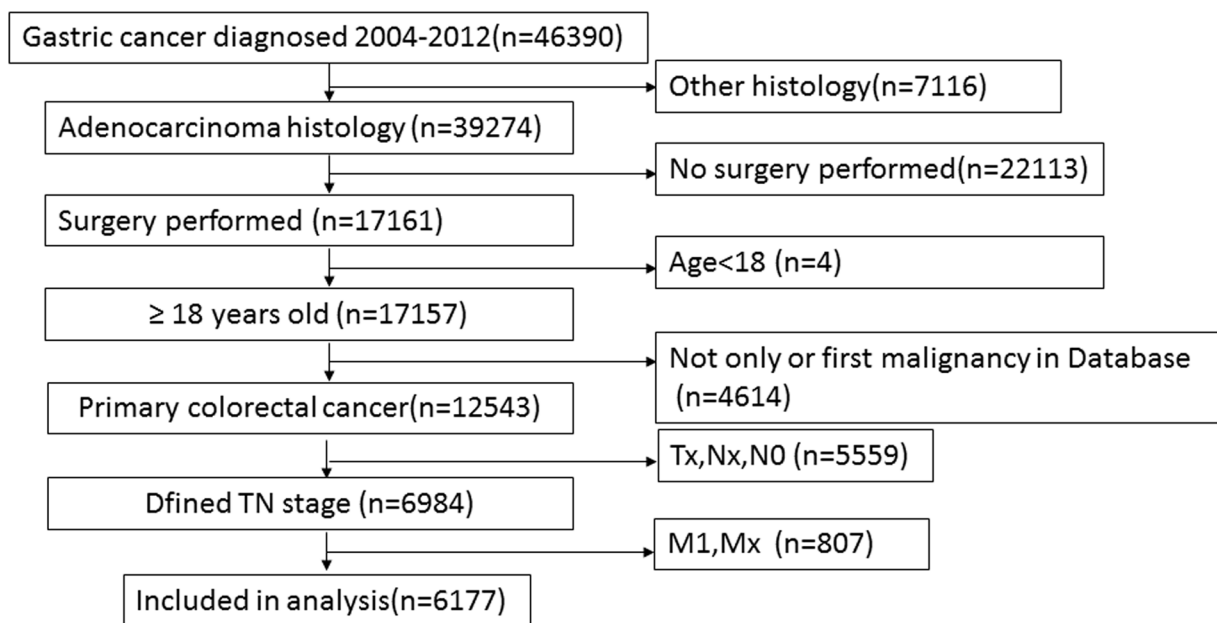

Supplementary Figure S1: Flow chart of patients' cohort (N=6, 177) definition.

Supplement: Supplementary file 1 [file oncotarget-07-35084-s001.pdf]
